# A Review on the Application of Nanocellulose in Cementitious Materials

**DOI:** 10.3390/nano10122476

**Published:** 2020-12-10

**Authors:** Aofei Guo, Zhihui Sun, Noppadon Sathitsuksanoh, Hu Feng

**Affiliations:** 1 Civil and Environmental Engineering Department, University of Louisville, Louisville, KY 40292, USA; a0guo003@louisville.edu (A.G.); z.sun@louisville.edu (Z.S.); 2 Chemical Engineering Department, University of Louisville, Louisville, KY 40292, USA; n.sathitsuksanoh@louisville.edu; 3 School of Civil Engineering, Zhengzhou University, Kexue Avenue 100#, Zhengzhou 450001, China

**Keywords:** nanocellulose, cementitious material, cellulose nanocrystal, cellulose nanofibril, bacterial cellulose, cellulose filament

## Abstract

The development of the concrete industry is always accompanied by some environmental issues such as global warming and energy consumption. Under this circumstance, the application of nanocellulose in cementitious materials is attracting more and more attention in recent years not only because of its renewability and sustainability but also because of its unique properties. To trace the research progress and provide some guidance for future research, the application of nanocellulose to cementitious materials is reviewed. Specifically, the effects of cellulose nanocrystal (CNC), cellulose nanofibril (CNF), bacterial cellulose (BC), and cellulose filament (CF) on the physical and fresh properties, hydration, mechanical properties, microstructure, rheology, shrinkage, and durability of cementitious materials are summarized. It can be seen that the type, dosage, and dispersion of nanocellulose, and even the cementitious matrix type can lead to different results. Moreover, in this review, some unexplored topics are highlighted and remain to be further studied. Lastly, the major challenge of nanocellulose dispersion, related to the effectiveness of nanocellulose in cementitious materials, is examined in detail.

## 1. Introduction

Nanomaterials, such as nano-silica [1,2], nano-TiO_2_ [3,4], and carbon nanotubes (CNTs) [5,6], have been widely used in cementitious materials. However, with the development of our society, more and more attention from researchers is given to renewable and sustainable nanomaterials. In response, nanocellulose as the nano-structured cellulose on the earth offers an alternative, which can be obtained from many cellulose sources, such as plants, animals, and bacteria [7]. Because of its strong, light, renewable, abundant, and environmentally friendly characteristics, nanocellulose has been widely used in many fields, such as food, cosmetics, and pharmaceuticals [8,9,10]. Until recent years, more and more researchers started to expand nanocellulose application in cementitious materials [11,12,13,14,15].

In cementitious materials, mainly four types of nanocellulose, including cellulose nanocrystal (CNC), cellulose nanofibril (CNF), bacterial cellulose (BC), and cellulose filament (CF) have been used [16,17]. The CNC, CNF, and CF are obtained from plants. Whereas BC is obtained from bacteria (such as *Gluconacetobacter xylinus*). Although these nanocelluloses are different from their origins and isolation methods, they have some similarities, such as low density, large aspect ratio, high strength, and high surface area, etc., [18,19,20,21,22,23,24]. Also, nanocelluloses are hydrophilic, with large amounts of hydroxyl groups on their surface, and can absorb some water while mixing with cementitious materials. Even they can be chemically functionalized to meet specific needs by modifying the hydroxyl groups and tuning the degree of hydrophilicity [25]. Based on these properties, nanocellulose may bring significant impacts on the properties of cementitious materials.

Nanocellulose has been reported to change the properties of cementitious materials significantly, and it has the potential to be used for different purposes [16,17]. First, nanocellulose can be used to improve the mechanical properties of cementitious materials if a proper dosage is used [15,20,26,27]. However, it should be noted that if a high dosage of nanocellulose is used, the aggregation of nanocellulose may occur, which can impair the mechanical properties. Therefore, under such conditions, a dispersion method (e.g., sonication) needs to be applied to nanocellulose to improve its effect on the mechanical properties [28]. Second, nanocellulose can be used as a type of viscosity-modifying agent (VMA) in cementitious materials. Hisseine et al. reported that cellulose filaments (CFs) could improve the mixture stability in self-consolidating concrete (SCC) by increasing the yielding stress and plastic viscosity of SCC [11]. Zhang et al. suggested that cellulose nanofibrils (CNFs) could improve the stability of cement pastes with a high w/c ratio to allow them to hydrate without settling during chemical shrinkage measurements [29]. Third, nanocellulose with a proper dosage can reduce the shrinkage of cementitious materials, especially with a low water-to-cement (w/c) ratio [13,14,19]. Similar to the effect on mechanical properties, the aggregation of nanocellulose caused by a high dosage may result in a negative result (increased shrinkage). In addition, nanocellulose has many other applications, such as improving the ductility of strain-hardening cementitious composites [12].

To help researchers learn about the application of nanocellulose in cementitious materials, there is a need to summarize the existing research. This state-of-the-art review first introduces the origin of nanocellulose, namely cellulose, and the characteristics of four types of nanocellulose (CNC, CNF, BC, and CF) that have been used in cementitious materials. Then the effects of these four types of nanocellulose on the properties of cementitious materials are introduced in detail. Lastly, the biggest challenge (nanocellulose dispersion issue) during the application of nanocellulose in cementitious materials is discussed. It is believed that the comprehensive summary of the application of nanocellulose in cementitious materials can lay a foundation and give insights for future investigations.

## 2. Nanocellulose

### 2.1. Origin of Nanocellulose (Cellulose)

**Figure 1:**
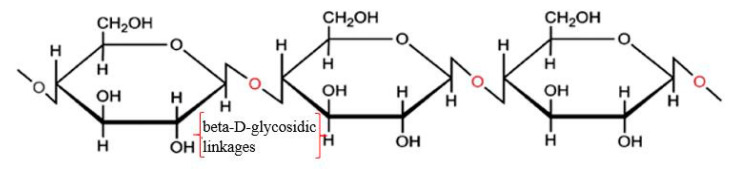
Structure of cellulose. Reproduced from [32], with permission from Elsevier, 2015.

Nanocellulose can be obtained from cellulose through a series of chemical or physical treatments. Cellulose is a semi-crystalline polysaccharide [(C_6_H_12_O_5_)_n_] consisting of D-glucopyranose units joined by β-(1-4)-glycosidic linkages (shown in Figure 1), and the degree of polymerization can be around 10,000 [30,31,32]. Cellulose can be extracted from plants (e.g., hemp, flax, and jute) and woods and also can be synthesized by algae, tunicate, and bacteria [33]. It exists in seven allomorphs (cellulose I_α_, I_β_, II, III_I_, III_II_, IV_I_, and IV_II_), of which cellulose I has the most crystalline structure with the highest axial elastic modulus [34]. Cellulose I_β_ is more thermally stable than cellulose I_α_ because of the weaker hydrogen bond in cellulose I_α_. Cellulose includes not only tightly packed slender rod-like crystalline microfibrils but also amorphous regions that have a lower packing density [31,32]. Because most of the cellulose is crystalline, it is not easy to be broken down [35]. It was reported that cellulose is resistant to strong alkali and oxidizing agents but is easily hydrolyzed by acid to water-soluble sugars [36]. The decomposition of cellulose can produce a monosaccharide called glucose (e.g., C_6_H_12_O_6_), which includes three hydroxyl groups, with two of them forming intramolecular hydrogen bonds and one of them forming intermolecular hydrogen bonds [37].

### 2.2. Classification of Nanocellulose

In cementitious materials, the commonly used nanocelluloses include: (1) cellulose nanocrystal (CNC), also referred to as nanocrystalline cellulose (NCC) and cellulose nanowhisker (CNW); (2) cellulose nanofibril (CNF), also referred to as nano-fibrillated cellulose (NFC); (3) bacterial cellulose (BC); and (4) cellulose filament (CF). The characteristics of each form are reviewed as follows.

#### 2.2.1. Cellulose Nanocrystal (CNC)

**Figure 2:**
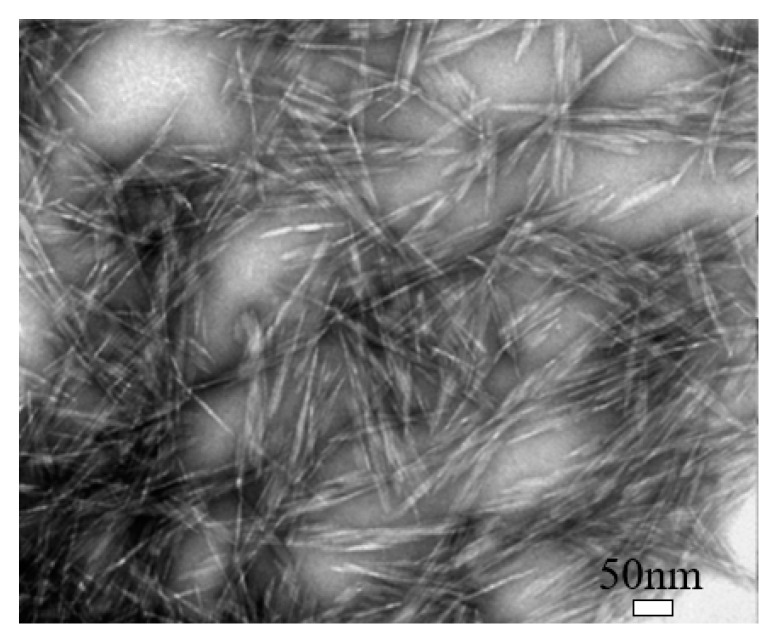
Transmission electron microscopy (TEM) image of cellulose nanocrystal (CNC). Reproduced (adapted) from [38], with permission from American Chemical Society, 2013.

Cellulose nanocrystal (CNC) is a type of cellulose-based nanomaterials, which is rod-like or whisker-shaped, with a width of 3–20 nm and a length of 50–2000 nm, as shown in Figure 2 [38]. It contains 64–98% cellulose I_β_ depending on the source [34]. Normally, it can be produced using different methods, of which the hydrolysis with mineral acids, especially sulfuric acid (H_2_SO_4_), is the most commonly used, and the phosphoric acid (H_3_PO_4_) and hydrochloric acid (HCl) are also used [22]. The acid treatment can remove most amorphous cellulose and thus produce high purity cellulose crystals, leading the CNC to have a high crystallinity [38]. Also, during the sulfuric acid hydrolysis process, some negatively charged sulfate ester groups can be grafted on CNC’s surface [39]. CNC possesses some unique properties, such as high crystallinity, relatively high aspect ratio (10–100), high thermal stability (up to 300 °C), low density (~1.6 g/cm^3^), low coefficient of thermal expansion (~1 ppm/K), large surface area, high tensile strength (~7.5 GPa), and high tensile modulus (up to 170 GPa), and even it can be easily functionalized because of easily accessible hydroxyl groups on its surface [22,23,24,25].

#### 2.2.2. Cellulose Nanofibril (CNF)

**Figure 3:**
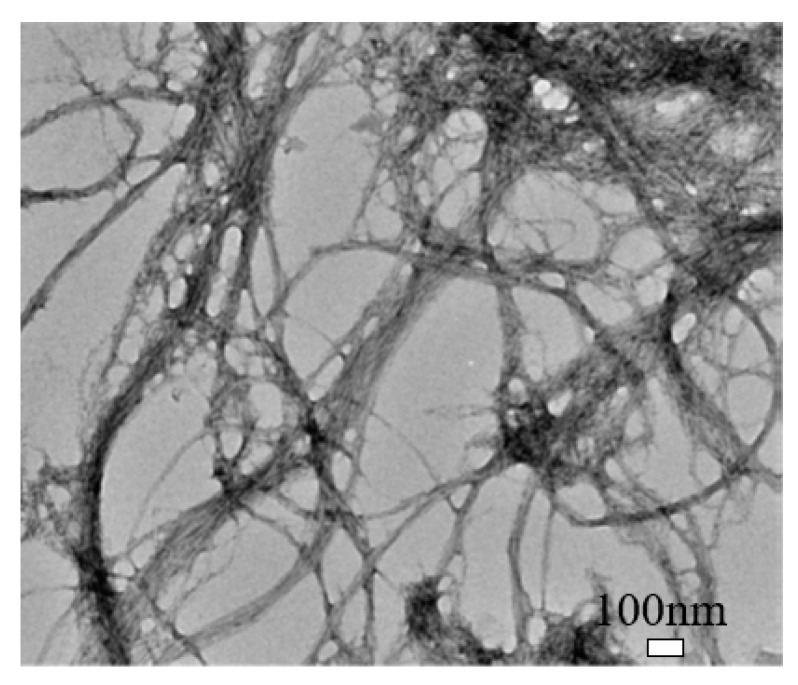
Transmission electron microscopy (TEM) image of cellulose nanofibril (CNF). Reproduced (adapted) from [38], with permission from American Chemical Society, 2013.

Cellulose nanofibril (CNF) is different in shape, size, and composition from CNC, as shown in Figure 3 [38]. It exhibits a complex, highly entangled, and web-like structure. The entanglement and percolation of CNF can increase the probability of fiber agglomeration compared to CNC [38]. CNF typically has a width of 50 nm and a length of less than 0.2 mm [19]. It has a high aspect ratio, low density, and high specific surface that can enable functionalization. Also, it has more amorphous cellulose and is less crystalline than CNC. CNF can be produced by TEMPO-mediated oxidation, multi-pass high-pressure homogenization, enzymatic hydrolysis, and direct mechanical fibrillation [38,40,41].

#### 2.2.3. Bacterial Cellulose (BC)

**Figure 4:**
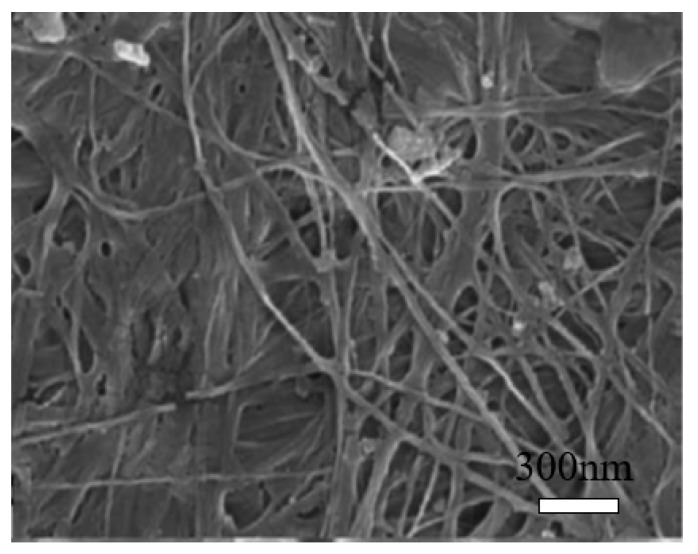
Scanning electron microscopy (SEM) image of bacterial cellulose (BC). Reproduced from [45], with permission from Elsevier, 2015.

Bacterial cellulose (BC) can be produced from many bacterial genera, such as Acetobacter, Achromobacter, Aerobacter, and Agrobacterium, etc. The most commonly used bacterial strain is Gram-negative bacteria, *Gluconacetobacter xylinus*, previously known as *Acetobacter xylinum* [42,43]. The production of bacterial cellulose consists of two important processes, fermentation and purification. During the fermentation process, the microorganisms can move freely in the medium or attach to the cellulose fibers, leading to a highly swollen gel structure [44]. The purification process involves the death of microorganisms and the removal of cell wastes and culture medium from the cellulose matrix [44]. The morphology of BC is shown in Figure 4 [45]. It may change depending on specific bacteria and culturing conditions [46]. BC is an organic compound with the chemical formula of (C_6_H_10_O_5_)_n_, which is identical to the plant cellulose. However, BC has higher crystallinity, polymerization degree, purity, water absorption capacity, and tensile strength [20].

#### 2.2.4. Cellulose Filament (CF)

**Figure 5:**
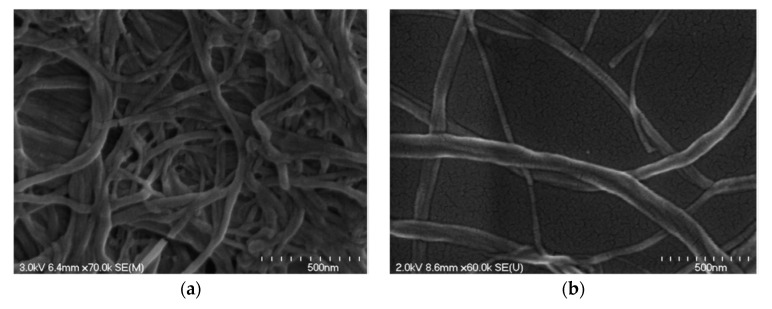
Scanning electron microscopy (SEM) images of (**a**) cellulose filament (CF) network and (**b**) individual CF fibril. Reproduced from [21], with permission from American Society of Civil Engineers, 2018.

Cellulose filament (CF) is a mechanically processed cellulose fibril, without any chemical or enzymatic treatment, and the fully mechanical process can “peel” fibers longitudinally, thus preserving their initial length as much as possible, while reducing their diameter by approximately 1000-fold [47]. The morphology of the CF network and individual CF fibril can be seen in Figure 5a and b, respectively [21]. It has a similar structure to CNF, but it may have a significantly higher aspect ratio (100–1000), with a length of around 100–2000 μm and a diameter of 30–400 nm [21].

## 3. Application of Nanocellulose in Cementitious Materials

The effects of different types of nanocellulose (CNC, CNF, BC, or CF) on the properties of cementitious materials are different. Here we discuss their effects on the physical and fresh properties, hydration of cement, mechanical properties, microstructure, shrinkage, rheology, and durability of cementitious materials.

### 3.1. Cellulose Nanocrystal (CNC) in Cementitious Materials

#### 3.1.1. Physical and Fresh Properties

Barnat-Hunek et al. examined some physical properties of concrete with cellulose nanocrystals (CNCs) and concluded that CNCs could not only reduce the water absorption but also reduce the wetting and adhesion properties as confirmed by the increased contact angle and the reduced surface free energy [27]. In another study conducted by Barnat-Hunek et al., the water absorption coefficient of mortar was reduced as the amount of CNCs increased, and 1.5% CNCs by weight of cement could lead to a reduction of 32% compared to the reference [48].

In terms of density, Barnat-Hunek et al. reported that with the increase of CNC dosage from 0.5% to 1.5%, the bulk density and specific density could be increased, with the former exhibiting a larger enhancement [27,48]. In Vanin et al.’s study, with the increase of CNC dosage from 0.1% to 1% by volume of cement, the bulk density was first increased, reaching a maximum value at 0.1% CNCs, and then gradually decreased until below the reference [49]. Meanwhile, the apparent density decreased as the CNC dosage increased, which was attributed to the CNC agglomeration, which held water molecules together, and the entrapped air, which led to higher capillary pores.

#### 3.1.2. Hydration of Cement

Cement hydration is a very important chemical process that governs the performance of cementitious materials. Since cement hydration is an exothermic reaction, the isothermal calorimetry (IC) analysis is often conducted to evaluate the hydration process through monitoring the heat evolution and the heat evolution rate. The hydration behavior of CNC-reinforced cement paste was studied by Cao et al. [50], and the IC test indicated that the peak heat flow was delayed with the addition of CNCs. In contrast, the degree of hydration (DoH) at a given age (e.g., 7 day) is increased. Similar conclusions were obtained by Flores et al. who indicated that even if the size of CNCs used is larger than that used by Cao et al. [50], the overall trend in the hydration heat is similar to each other, and CNCs were observed to delay cement hydration at an early age but improve the hydration at a later age [24]. Besides, Ghahari et al. indicated that the use of 0.2% and 1% CNCs by volume of cement paste could extend the dormant period of cement hydration that is indicative of the delayed hydration but increase the hydration heat that is indicative of the increased degree of hydration [51].

**Figure 6:**
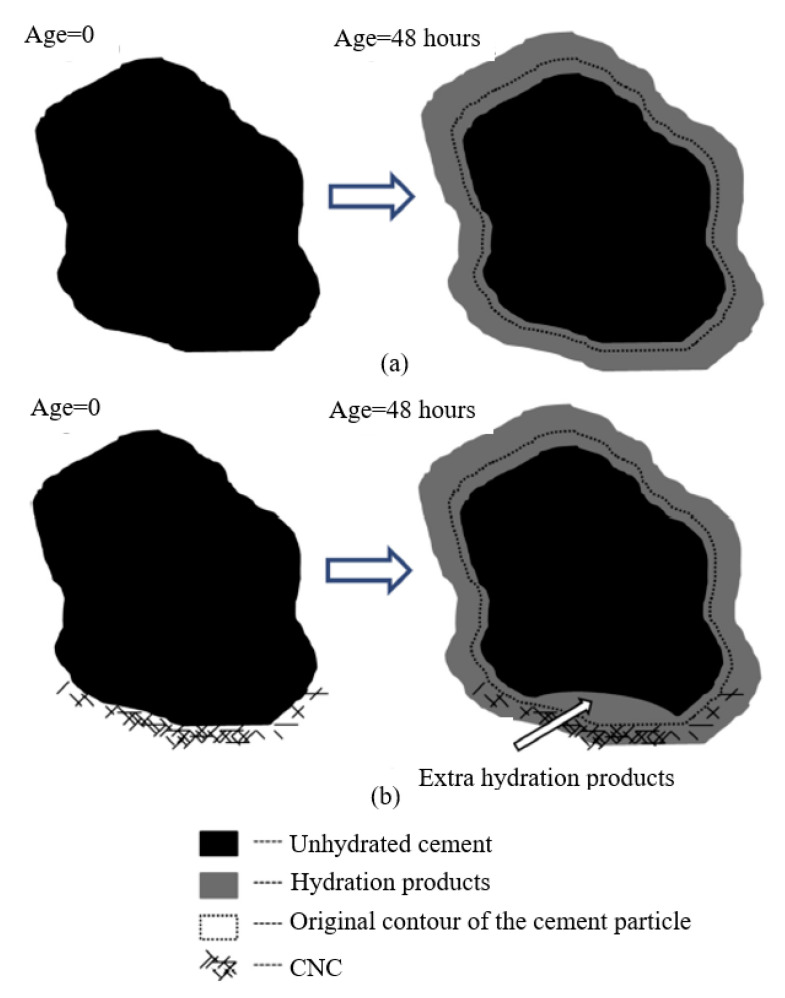
Schematic illustration of hydration products forming around cement particles for (**a**) plain cement pastes and (**b**) cement pastes with CNCs. Reproduced from [50], with permission from Elsevier, 2015.

The delaying effect on the cement hydration (specifically the initiation of hydration) is mainly because CNCs adhere to cement particles and block the cement particles from reacting with water. The increased degree of hydration (DoH) at a given age can be attributed to two possible reasons [50]. One reason is steric stabilization that is commonly observed in some water-reducing admixture (WRA), by which CNCs can disperse cement particles more uniformly and thus enhance the reaction efficiency with water. However, the degree of hydration analysis on cement pastes with WRA indicates that the steric stabilization does not improve the DoH significantly, which indicates that the steric stabilization may not be the dominant reason. The other reason is short circuit diffusion (SCD), as schematically shown in Figure 6. For plain cement pastes (Figure 6a), during cement hydration, the hydration products can form a shell around the unhydrated cement particles, which can prevent water from diffusing into the interior unhydrated cement particles and thus slow down the hydration rate. However, for cement pastes with CNCs (Figure 6b), the CNCs initially adhere to cement particles and remain inside the hydrated cement particles as the hydration progresses. The CNCs can provide some channels for water transportation through hydration products to inner unhydrated cement particles, which can improve cement hydration. Moreover, the short circuit diffusion mechanism is confirmed to be more dominant than the steric stabilization mechanism.

CNCs from various raw material sources and processing techniques can affect cement hydration differently when cement type varies. Fu et al. applied nine different CNCs to cement pastes made using Type I/II cement and Type V cement, respectively [52]. One important finding was that even if all CNCs could increase the degree of hydration at a given age, compared to some CNCs obtained by acid hydrolysis, the CNCs obtained by transition metal-catalyzed oxidation was particularly effective in increasing hydration heat release regardless of the cement type. The other important finding was that the IC test showed that at an early age, the retardation effect (a delay in the time to reach peak heat flow) on cement hydration in Type V cement paste was more pronounced than that in Type I/II cement paste; however, the total heat release in Type V cement paste was greater. This may be due to a relatively higher aluminate phase content in the Type I/II cement. The hydrated tricalcium aluminate (C_3_A) phases (ettringite and monosulfate) tend to adsorb more CNCs than other hydrated phases, leaving fewer CNCs to adhere to the silicate phases in the Type I/II cement paste, which results in less retardation effect on cement hydration. Meanwhile, fewer CNCs on the surface of cement particles can be available for the short circuit diffusion (SCD) effect, resulting in a lower degree of hydration at a given age. Therefore, it can be seen that the effectiveness of CNCs relies on the C_3_A content in cement.

#### 3.1.3. Mechanical Properties

The mechanical properties of cementitious materials with CNCs are summarized in Table 1. Several conclusions can be drawn. First, a modest dosage of CNCs (e.g., 0.2% by weight of cement) can improve the mechanical properties, whereas a high dosage of CNCs can result in a negative effect [13,15,49,50,51,53,54]. The improvement in the mechanical properties of cementitious materials at low dosages of CNCs can be attributed to the increased degree of hydration at a given age. Cao et al. examined the relationship between the flexural strengths at the age of 3 and 7 days and the degree of hydration (DoH) data from isothermal calorimetry and found that at low dosages of CNCs, the flexural strength increased nearly linearly as a function of DoH [50]. Therefore, the improvement in mechanical properties can be closely related to the increase in DoH. Moreover, by comparing with water-reducing agents (WRA), Cao et al. showed that the CNCs were more effective in improving the flexural strength than WRA [50]. Because the hydration analysis indicated that the steric stabilization mechanism does not dominate the increase of the degree of hydration, the WRA cannot improve the flexural strength only by steric stabilization. Besides, Cao et al. suggested that CNCs had the potential to combat microcracking and thus improve the mechanical performance of cementitious composites [55]. At high dosages of CNCs, the reduction in the mechanical properties can be attributed to the aggregation of CNCs that can act as stress concentrators and damage the mechanical properties of cementitious materials [50].

Second, the effect of CNCs on mechanical properties also depends on the cement type. Claramunt et al. showed that 0.1% and 0.2% CNCs could improve the modulus of rupture and modulus of elasticity of calcium aluminate cement (CAC) paste; however, this was not observed in ordinary Portland cement (OPC) paste [54]. As introduced in another research [52], the effectiveness of CNCs relied on the aluminate phase (C_3_A content) in cement, and a higher aluminate phase could reduce the degree of hydration (at a given age) at the same dosage of CNCs. As such, it is expected that at the same CNC dosage, the CAC with a high aluminate phase can have a lower degree of hydration than OPC at a given age. However, Claramunt et al. reported that the CNCs could improve CAC’s mechanical properties more than those of OPC paste [54]. Therefore, the difference in mechanical properties cannot be attributed to the degree of hydration, and some other factors, such as pore structure, may dominate. Claramunt et al. reported that in the CAC samples, the CNCs could act as a scaffold for the precipitation of hydration products, which could connect pore walls and give continuity to the matrix while maintaining the matrix’s porosity, resulting in better mechanical properties. However, no detailed comparison was made with OPC samples. More research need to be done on this topic.

Third, the addition of a modest dosage (e.g., 0.2% by weight of cement) of CNCs can improve cementitious materials’ mechanical properties (compared to the reference) at early ages more than those at later ages. Vanin et al. studied the dynamic modulus of elasticity of cement pastes with varying dosages of CNCs through the impulse excitation technique and found that low dosages of CNCs (0.1%, 0.2%, and 0.5% by volume of cement) could increase the dynamic modulus of elasticity of cement pastes at early ages (up to 14 days). In contrast, they do not have a significant influence at later ages (28 days) [49]. In addition, Dousti et al. showed that the addition of 0.5% CNCs (by weight of cement) to oil well cement paste could accelerate the compressive strength gain, and the 1-day compressive strength of cement pastes with CNCs could be over 2.5 times higher than that of the paste without CNCs [56]. However, at 56 days, even if the difference is still around 30%, the improvement is less significant. The tensile strength showed a similar trend. The use of CNCs can promote early strength development that is desired in oil well cementing.

#### 3.1.4. Microstructure

**Figure 7:**
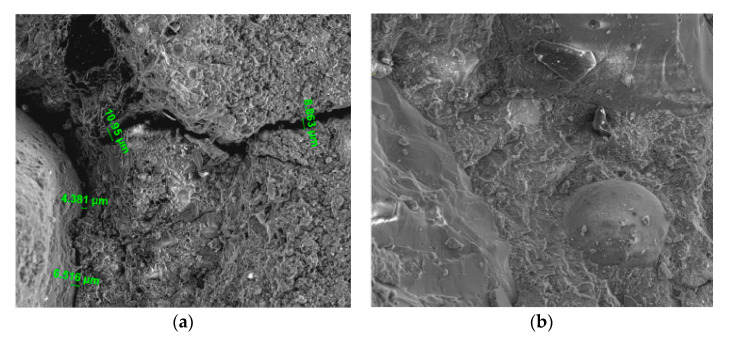
SEM images of concrete (**a**) without and with (**b**) 1% CNCs (1000× magnification). Reproduced from [27], with permission from Elsevier, 2019.

The use of CNCs can affect the pore structure of cementitious materials significantly. The pore size analysis by nitrogen adsorption showed that the addition of CNCs could refine the pore structure of the cement paste [57]. Barnat-Hunek et al. indicated that adding CNCs into concrete could reduce the number of pores, and especially 1% CNCs (by weight of cement) could result in significantly fewer micro-pores and micro-cracks than the plain concrete, as shown in Figure 7 [27]. Moreover, Dousti et al. indicated that even if adding CNCs to oil well cement paste did not change the mean pore size appreciably (at least not in the range of pore size <100 nm), the overall porosity was reduced by 40% for pores <100 nm in size [56]. Therefore, reducing the porosity and refining the pore structure can be achieved by CNCs.

CNCs can also help modify the microstructure of hydration products. The statistical nanoindentation measurements indicated that the volume fraction of high-density C-S-H was increased slightly, and the volume fraction of low-density C-S-H was decreased for cement mixtures with CNCs compared to the control mixture [24]. Cao et al. indicated that the addition of CNCs exerted little effect on the reduced modulus of unhydrated cement and low-density C-S-H areas because most CNCs lied in high-density C-S-H regions [58]. In high-density C-S-H regions, the reduced modulus was enhanced significantly even if the hardness was hardly affected. There are two possible reasons for this improvement: CNCs have a high elastic modulus, which is higher than that of the high-density C-S-H; and the modulus of high-density C-S-H area may be changed because its structure or chemistry may be modified by CNCs. Meanwhile, some cracks were observed on SEM images, and cracks could pass through the interfaces between unhydrated cement particles and the high-density C-S-H or between the low- and high-density C-S-H regardless of CNCs concentrations.

#### 3.1.5. Shrinkage

Lee et al. studied the drying shrinkage of fiber (flax and steel)-reinforced cement composites with CNCs and found that the drying shrinkage was reduced for all specimens containing CNCs, and a 55% reduction was observed when CNCs were used at a dosage of 0.8% by volume of cement [13]. The reduction in drying shrinkage might be because CNCs promoted cement hydration and increased the number of ettringites, resulting in expansion, which led to reduced shrinkage.

#### 3.1.6. Rheology

Montes et al. studied the rheological properties of cement pastes (Type I/II cement and Type V cement, respectively) with nine types of CNCs from various sources and found that there was no evidence to show that the length, the aspect ratio, and the zeta potential of CNCs were deterministic factors that can influence the yield stress of the paste mixture [59]. However, varying dosages of CNCs could result in different effects. When the CNC dosage was below a specific dosage (0.2% in most cases), all CNCs could reduce the yield stress of the paste mixture, and beyond this dosage, CNCs could increase the yield stress. Similarly, Cao et al. did a rheological analysis on cement pastes with CNCs and found that the yield stress initially decreased with CNCs when the dosage of CNCs was low (<0.3% by volume of cement) and then switched to increase when the dosage of CNCs was large (>0.3%) [50]. The initial reduction of yield stress can be attributed to the steric stabilization that improves the dispersion of cement paste. The latter increase of yield stress may be due to the aggregation of CNCs or the similar function to a viscosity-modifying admixture (VMA) [50,59].

#### 3.1.7. Durability

The electrical resistivity measurement could evaluate the durability performance of cement-based materials. Compared to the reference, a slight improvement in the electrical resistivity was observed when CNCs were incorporated into the cement mixtures [24]. In addition, Barnat-Hunek et al. concluded that CNCs could increase the freezing-thawing resistance of concrete, with 1% CNCs (by weight of cement) leading to better improvement than 0.5% CNCs [27]. In their study [48], the resistances to the freezing-thawing and the detrimental effect of salt were also reported to increase with the addition of CNCs. However, Lee et al. showed that at an optimal CNC dosage of 0.8% by volume of cement, even if the strength of fiber-reinforced high-toughness cement composites could be improved, the resistance to freezing-thawing was reduced slightly, which could be attributed to the fact that CNCs reduced the voids of composites and then increased the expansion pressure during freezing-thawing [53]. On the bright side, CNCs could reduce the carbonation depth and chloride ion penetration [53].

### 3.2. Cellulose Nanofibril (CNF) in Cementitious Materials

#### 3.2.1. Physical and Fresh Properties

Cellulose nanofibrils (CNFs) are hydrophilic because of the many hydroxyl groups, which can absorb much water in cementitious materials. Therefore, it was widely reported that with the increase of CNF dosage, the flowability of cementitious materials was decreased [19,60]. Haddad Kolour et al. even showed that every 0.05% of additional CNFs incorporated in cement pastes decreased the flow value by almost 15% while increasing CNF dosages from 0.015% to 0.15% by weight of cement [14].

Barnat-Hunek et al. examined the water absorption of concrete with CNCs and CNFs, respectively [27]. Both of them could reduce the water absorption and the wetting and adhesion properties of concrete, and even the reduction caused by CNFs seemed to be less than CNCs did.

In addition, the thermal conductivity and thermal expansion of paste mixtures with CNFs were studied by Mejdoub et al. [61]. With the increase of CNF dosage up to 0.3% by weight of cement, the thermal conductivity and thermal expansion of the paste mixtures were increased, which was attributed to the densification of cement matrix as confirmed by the reduced porosity and increased bulk density [61]. However, when the CNF dosage was beyond 0.3%, the addition of CNFs could reduce the thermal conductivity, as confirmed by the increased porosity and reduced bulk density due to fiber agglomeration.

#### 3.2.2. Hydration of Cement

Similar to CNCs, many researchers reported that CNFs could delay the initiation of cement hydration [60,62,63]. For example, in the study conducted by Jiao et al. [60], it was found that although no significant difference could be seen for samples with and without CNFs at 10 h, the addition of CNFs could lengthen the induction period and delay peak heat flow of cement pastes at later ages. The delaying could be because the hydroxyl group and the carboxyl group of CNFs reacted with Ca^2+^ in cement pastes forming some hydrophilic complexes to adhere to cement particles’ surface and thus slowed down the formation rate of calcium silicate hydrate (C-S-H) and calcium hydroxide (CH). This explanation is similar to that given for CNCs, and the adherence of CNFs or CNCs to cement particles is the main mechanism. In addition, Onuaguluchi et al. explained the delaying effect of CNFs that highly viscous nanofiber gels could delay the dissolution of cement and reduce the mobility of cement particles, and thus slow down the formation rate of hydrates [62].

On the other hand, the degree of hydration (DoH) of cement at a given age (e.g., 7 days) can be increased by CNFs [60,62,63]. An explanation for the increase of DoH at the same w/c ratio is that the CNFs allows for a more efficient reaction between cement particles and water and improves the uniformity of cement particle-water mixture, reducing the amount of unhydrated particles [60]. The explanation is similar to the steric stabilization effect reported by Cao et al. [50]. However, in the application of CNCs, Cao et al. indicated that the improvement in the DoH by the steric stabilization was not significant, and the short circuit diffusion might be the main mechanism [50]. Haddad Kolour et al. suggested that incorporating CNFs with dosages from 0.015% to 0.15% by weight of cement into cement pastes could improve the 3-day degree of hydration by up to 8%, which could be attributed to the short circuit diffusion and even the internal curing effect [14]. Lastly, there are two other possible explanations to account for the increased DoH. One is that the alkaline hydrolysis of CNFs releases heat and may increase cement hydration [62]. The other is CNFs can act as nuclei to promote the formation of hydration products [61].

Given the delaying effect of CNFs on the initiation of cement hydration, it is expected that the setting time of cement pastes may be increased by CNFs. Without any doubt, Jiao et al. reported that the initial and final setting times of cement pastes increased with the increase of CNFs from 0.05% to 0.40% by weight of cement [60]. However, Mejdoub et al. reported that CNFs at dosages from 0.01% to 0.5% by weight of cement could reduce the initial and final setting times [61]. The CNFs seemed to act as a set accelerator to result in a faster hydration process. The opposite results from these two studies may be dominated by the characteristics of CNFs because Zhang et al. pointed that the chemical constitutions other than homopolysaccharides might be different for different sources of CNFs, which could have various effects on cement hydration [29].

#### 3.2.3. Mechanical Properties

The effect of CNFs on the mechanical properties of cementitious materials is summarized in Table 2. Several conclusions can be elaborated based on the summary. First, same as CNCs, in most cases, a modest dosage of CNFs can increase the mechanical properties, and yet too high CNF dosage can decrease the mechanical properties [19,54,60,61,62,63]. There are several possible reasons for the improved mechanical properties [60,61,62,63]: (1) CNFs increases the degree of hydration at a given age, promoting the formation of large volumes of hydration products; (2) high specific surface area of CNFs improves the bonding between CNFs and the matrix, which assures the improvement of stress transfer; (3) high hydrophilicity of CNFs promotes adhesion to cement pastes; (4) CNFs can link different parts of hydrated cement by bridging effect and thus prevent crack propagation [14,64]; (5) CNFs can serve as internal curing agents to retain water at early ages and release it later [14,19]. From the microscopic point of view, the reason can be that the addition of CNFs can improve the microstructure of cementitious material [61,65]. For the decreased mechanical properties at a high dosage of CNFs, the reason is that a high dosage of CNFs can aggregate and lead to nonuniform distribution, which results in stress concentration and then decreases the mechanical properties [14,19,60,61,62,63].

Second, contrary to the above-mentioned changes caused by CNFs, some researchers report a consistent decrease in the mechanical properties of cementitious materials with an increase of CNF dosage. For example, Goncalves et al. reported that 0.1–0.5% of dry CNFs by volume of mortar could reduce the 28-day compressive strength of mortar (at a w/c ratio of 0.485 and after moist curing) by 9.7% on average, and no significant difference was observed while the dosage of CNF varies in that range [66]. Similarly, Kolour et al. reported that the addition of CNFs at dosages of 0.1, 0.2, and 0.3% did not have a positive effect on the compressive strength of concrete at a given w/c ratio (>0.5) [19]. Besides, the change of compressive strength was less sensitive to w/c ratio when CNFs were incorporated. This may be because, at higher w/c ratios, the additional porosity introduced by the additional water no longer controls the compressive strength, and instead, the change in microstructure induced by the CNFs dominates.

Third, similar to CNCs, the effect of CNFs on the mechanical properties of cementitious materials depends on the cement type. In calcium aluminate cement (CAC) paste, the addition of 0.1% CNFs by weight of cement could increase the modulus of rupture (MOR) and modulus of elasticity (MOE); however, in ordinary Portland cement (OPC) paste, the improvement was not significant [54]. It was also noted that, in the CAC paste, if the CNF-reinforced pastes were subject to an accelerated aging process, 0.1% CNF did not result in any improvement, which could be due to the degradation of CNFs during the aging process.

Fourth, CNFs can result in a more pronounced effect on the mechanical properties at later ages than those at early ages. Jiao et al. reported that the CNFs did not have a significant effect on the 3-day compressive and flexural strengths but could improve the 7-day and 28-day strengths [60]. In particular, the 28-day compressive strength and flexural strength of cement paste with 0.15% CNFs could be improved by 15% and 20%, respectively. By comparing with the CNCs effect introduced in Section 3.1.3 in this review, it can be seen that in the existing research, CNFs (improve early strength less than later strength) and CNCs (improve initial strength more than later strength) exhibit the opposite effect. More research needs to be done to verify this phenomenon and reveal the mechanism.

Lastly, the effectiveness of CNFs in improving the compressive strength and flexural strength of cementitious materials is different from CNCs. It was reported that adding CNFs into concrete could improve its flexural strength to a greater extent than CNCs, and 1% CNFs and 1% CNCs by weight of cement could improve the flexural strength by 34.5% and 23.4%, respectively [27]. Meanwhile, CNFs could improve the compressive strength to a lower extent than CNCs, and 1% CNFs and 1% CNCs could improve the compressive strength by 23.3% and 37.9%, respectively. This may be due to the different structures of CNFs and CNCs, and CNFs are longer and have a lower crystallinity degree than CNCs.

#### 3.2.4. Microstructure

The addition of low dosages (e.g., 0.3 wt%) of CNFs into the cementitious matrix leads to a denser matrix with lower porosity [61]. Jiao et al. indicated that the pore size could be reduced with increasing CNF dosage, and also a modest dosage (0.15% by weight of cement) of CNFs could reduce the porosity of cement pastes from 13.9% to 13.5% [60]. The reduced porosity and pore size may be because a modest addition of CNFs can first absorb water and then release the water to promote cement hydration, which can produce more cement hydrates. However, when the dosage of CNFs reached 0.40%, the porosity of cement pastes was increased from 13.9% to 16.2% [60]. A similar result was reported by Goncalves et al. who showed that the addition of CNFs could increase the total porosity [65]. However, on the other side, the pore size of cementitious materials could be refined by CNFs. The porosity corresponding to the macropores (>10 μm in size) was reduced, and the porosity corresponding to the micro and mesopores (60 nm–10 μm in size) was increased. In Goncalves et al.’s another study [66], it was reported that adding CNFs to Type general use (GU) cement paste could increase the pores between 100 nm and 10 μm, which is consistent with the previous study. Meanwhile, the capillary pores in the range of 10–140 nm could be reduced.

**Figure 8:**
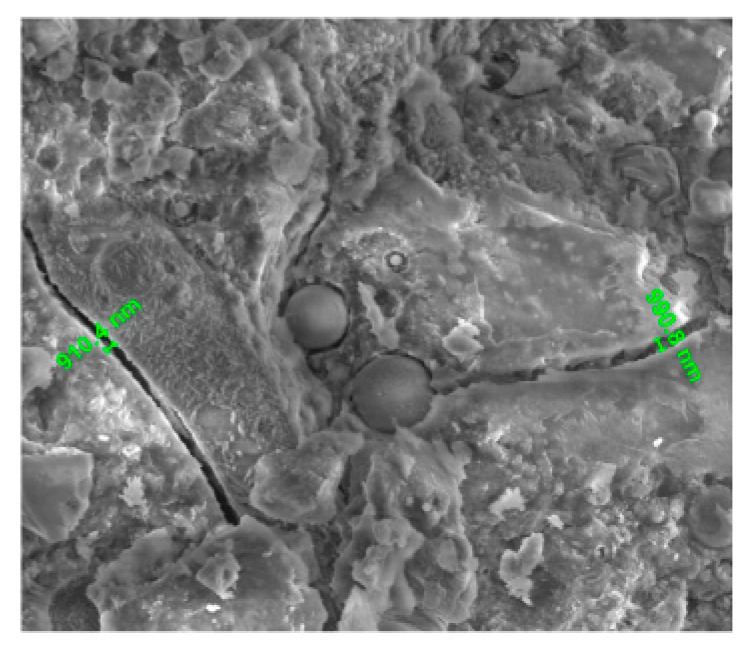
SEM image of concrete with 1% CNFs (5000× magnification). Reproduced from [27], with permission from Elsevier, 2019.

Also, Barnat-Hunek et al. compared the microstructures of concrete with CNFs and CNCs and indicated that concrete with CNFs had small pores and crack with up to 951 nm (shown in Figure 8), whereas concrete with CNCs was very compacted, and no pores and cracks could be found (shown in Figure 7b) [27]. CNFs could be worse than CNCs in reducing the micro-pores and micro-cracks.

#### 3.2.5. Shrinkage

Kolour et al. showed that a small dosage of CNFs (0.05, 0.1, 0.2, and 0.5% by volume of cement) could reduce free shrinkage of cement pastes at a low w/c ratio (w/c = 0.35), which is because a low w/c ratio could lead to a less porous cement paste, and the CNFs serving as internal curing agents could release water to minimize self-desiccation and thus reduce shrinkage [19]. However, a high dosage of CNFs (1.0, 1.5, and 3.0%) could increase the free shrinkage of pastes with a low w/c ratio (w/c = 0.35). Moreover, for pastes with high w/c ratios (0.40, 0.45, and 0.50), the free shrinkage of cement paste was not improved and even was more severe regardless of CNF dosage. Therefore, it can be seen that the effect of CNFs on the free shrinkage of pastes depends on both the CNF dosage and w/c ratio. Haddad Kolour et al. studied the autogenous shrinkage of cement pastes with a low w/c ratio (0.35) and indicated that adding 0.06% and 0.09% of CNFs by weight of cement could reduce the autogenous shrinkage up to 49% and 26%, respectively, which may be due to the internal curing effect [14]. Compared to the 0.06% dosage, the 0.09% dosage was less effective in autogenous shrinkage reduction, which may be due to the agglomeration of CNFs at a higher dosage. The results from this study are generally consistent with Kolour et al.’s results [19], and a common conclusion can be drawn that only when a low w/c ratio (e.g., 0.35) and a small dosage of CNFs are both satisfied, a reduced shrinkage can be seen. However, the CNF dosage boundary between low and high dosages may vary largely for different cementitious systems or different CNFs.

#### 3.2.6. Rheology

Hoyos et al. reported that CNFs could work as viscosity-modifying agents (VMA) to increase the yield stress of cement pastes exponentially as the CNF dosage increases from 0.1% to 0.4% by weight of cement [67]. Similarly, Sun et al. showed that with the increase of CNF dosage from 0.04% to 0.28% by weight of cement, the yield stress and plastic viscosity could be increased [63]. In Section 3.1.6 in this review, for cement pastes with CNCs, when the CNC dosage is low, an initial reduction in the yield stress can be observed because of the steric stabilization; however, the existing research for cement pastes with CNFs has not shown such a phenomenon. More research is needed to elucidate the mechanism of this phenomenon.

Since CNFs can be used as VMA, Zhang et al. created a stable gel using CNFs to support cement particles to allow them to hydrate without settling during the chemical shrinkage measurement for cement pastes with high w/c ratio [29]. In fact, for this application, one concern is that CNFs may change the hydration of cement. However, the results in this study indicated that CNFs did not change the cement hydration significantly for pastes with high w/c ratio, and the effect of CNFs on cement hydration is less than that of the w/c ratio. Therefore, CNFs can be used as VMA to help study the chemical shrinkage of cement pastes with a high w/c ratio.

#### 3.2.7. Durability

Goncalves et al. examined the sulfate penetration of mortars with four types of cement (general use cement, high sulfate resistance, high early strength, and blended cement with general use cement and fly ash) and concluded that the sulfate penetration could be decreased with CNF addition [65]. For example, for the general use (GU) cement mortar, a low dosage of CNFs (up to 0.2% volume fraction) could slightly reduce the sulfate penetration, and a high dosage of CNFs (0.3–0.4%) could reduce the sulfate penetration significantly. The study also proved that the expansion of mortars caused by sulfate attack was reduced by CNFs.

Goncalves et al. also examined the effect of CNF dosage (0.1% to 0.5% by volume of mortar) and the carboxyl group (-COOH, 0.13 to 1.13 mMol/g) grafted on the surface of CNFs on the resistance of cement mortar to chloride ingress [66]. A higher carboxyl group content was more favorable to the workability of mortar and could reduce the chloride ion penetration. For CNFs with 0.7 or 1.13 mMol/g carboxyl group, with the increase of its dosage, the chloride ion penetration of mortars could be reduced, which was most likely due to a lower porosity in the range of 10–140 nm in size. However, for CNFs with 0.13 mMol/g carboxyl group, when its dosage is not over 0.2%, the chloride ions of mortar could be reduced; beyond this dosage (0.2%), the chloride ion penetration could be increased, which might be because of the significantly reduced workability caused by CNFs.

Barnat-Hunek et al. concluded that the freezing-thawing resistance of concrete could be increased with the increase of CNFs, which was verified by the less pronounced mass loss and compressive strength decrease after 100 freezing-thawing cycles [27]. Compared to CNCs, the mass loss of concrete in the case of CNFs was less pronounced, whereas the compressive strength decrease was more significant.

### 3.3. Bacterial Cellulose (BC) in Cementitious Materials

#### 3.3.1. Physical and Fresh Properties

Bacterial celluloses (BCs) could be used in two different states, including gel and powder, and either form could reduce water absorption of cement mortar, with BC powder reducing more [20]. In general, the water absorption could be increased with the increase of BC dosage, which might be due to the aggregation of BCs.

#### 3.3.2. Mechanical Properties

For the mechanical properties of cementitious materials with BCs, the existing research is very limited, and only two papers can be found to date, as summarized in Table 3. BCs were used in two forms (powder and gel), and both forms were found to improve flexural strength (modulus of rupture), compressive strength, modulus of elasticity, and fracture toughness [20,45]. However, BC powders and BC gels have different effectiveness. Akhlaghi et al. indicated that generally, samples containing BC gels exhibited inferior mechanical properties compared to those containing BC powders [20]; however, Mohammadkazemi et al. indicated that BC powders could exhibit inferior mechanical properties compared to BC gels [45]. In addition, similar to CNCs and CNFs, the dosage of BCs can affect the mechanical properties of cementitious materials significantly. Akhlaghi et al. used BCs at dosages of 0.1, 0.3, and 0.5% by weight of cement in cement mortar and found that for flexural strength, the optimum dosages of BC powders and gels were 0.5% and 0.3%, respectively; for compressive strength, the optimum dosage of BC powders and gels were 0.3% and 0.1%, respectively. A high BC dosage could lower its efficiency, which could be attributed to its aggregation. Lastly, these two papers also provide an indirect way of using BCs to coat polypropylene fibers to improve the mechanical properties of fiber-reinforced mortar.

#### 3.3.3. Microstructure

**Figure 9:**
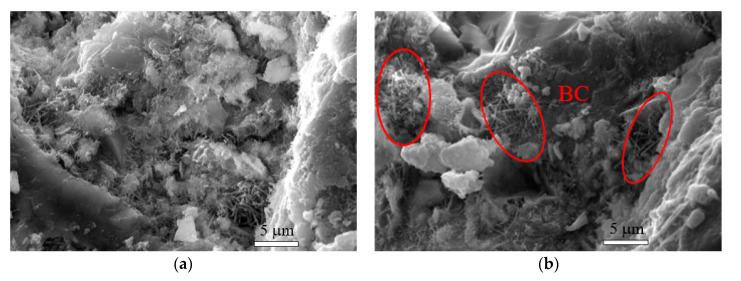

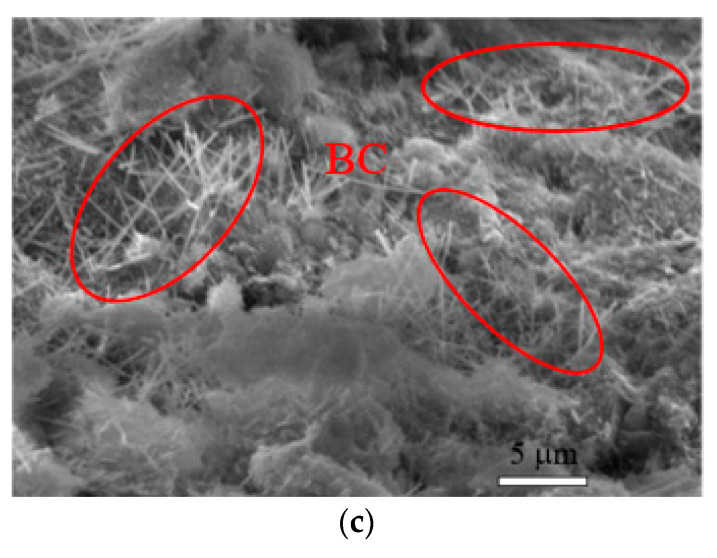
SEM images of (**a**) control specimen, (**b**) mortar with BC powder, and (**c**) mortar with BC gel. Reproduced from [20], with permission from Elsevier, 2020.

The interaction and interlocking between BCs and cement matrix could improve the anchoring, and BCs serve as nano-bridges to arrest nano-cracks to enable the load transfer between BCs and the matrix (shown in Figure 9) and thus improve the strength of cement paste [20]. When BCs were coated on the polypropylene fibers, the surface energy and roughness of fibers could be improved, which could increase the contact area between fibers and the matrix. Even the hydration products could accumulate on the surface of BC coated fibers, reducing the penetration of alkaline ions into the lumens of fibers and the fiber mineralization [45].

#### 3.3.4. Hydration of Cement, Shrinkage, Rheology, and Durability

The effect of BCs on the cement hydration, shrinkage, rheology, and durability of cementitious materials cannot be found in the existing research. Future studies are needed on these topics.

### 3.4. Cellulose Filament (CF) in Cementitious Materials

#### 3.4.1. Physical and Fresh Properties

The incorporation of cellulose filaments (CFs) could reduce the flowability of cementitious materials, and to maintain the same level of flowability, the demand for water-reducing admixture was increased with the increase of CFs [12,21,26]. This could be attributed to the hydrophilicity of CFs together with CF propensity for forming a percolating network due to high surface area and high aspect ratio. In addition, CFs were also found to increase the air content of ultra-high performance concrete (UHPC) [68].

#### 3.4.2. Hydration of Cement

The IC test indicated that the incorporation of CFs at dosages of 0.1%, 0.15%, and 0.2% by weight of paste mixture could slightly increase and broaden the silicate hydration peaks and also slightly increase the total released heat [21]. For example, 0.15% CFs in the paste mixture could increase the total released heat by 7% compared to the control. The increased hydration heat could be attributed to the hydrolysis of cellulose, which was an exothermic reaction and could promote cement hydration.

#### 3.4.3. Mechanical Properties

Up to date, the study about the application of cellulose filaments (CFs) in cementitious materials was only conducted by Hisseine et al. The CFs were provided by Kruger Biomaterials Inc. The mechanical properties of cementitious materials with CFs are summarized in Table 4. It was found that for cement pastes, the incorporation of CFs could increase the flexural strength, elastic modulus, and toughness, but it does not always improve the compressive strength significantly [21,26]. In a study [21], the CFs with a dosage of 0.1% to 0.2% could not increase the compressive strength, which might be due to the air entrainment caused by CFs. However, in another study [26], the CFs with a dosage of 0.05% to 0.30% could increase the compressive strength, which was attributed to the fact that the hydrophilic and hygroscopic CFs could dispatch supplementary water to the surrounding cementitious matrix to promote cement hydration. The authors attributed the difference in the effect of CFs on the compressive strength to the dispersion technique of CFs. The improved dispersion of CFs in the latter study might lead to the improvement of compressive strength.

Moreover, Hisseine et al. applied CFs to three different cementitious systems, self-consolidating concrete (SCC), strain-hardening cementitious composites (SHCC), and ultra-high performance concrete (UHPC), and CFs have various effects on these systems. In SCC, it was found that both compressive strength and flexural strength could be improved by CFs at dosages of 0.1%, 0.15%, and 0.2% by weight of binder [21]. Compared to the reduced compressive strength for cement pastes, the improvement in the compressive strength for the SCC system was because the aggregates in the SCC system could favor disintegrating CF clumps to promote CF dispersion and thus contribute to the compressive strength. In SHCC, it was found that CFs at dosages of 0.03%, 0.05%, and 0.1% by weight of cement was not pronounced in improving the first cracking strength and post cracking strength but was more pronounced in improving the ultimate strain capacity (in uniaxial tension) and the deflection capacity (in flexure) of SHCC [12]. In general, the CFs could be used to improve the ductility of SHCC. In the UHPC system, a 0.15% CF dosage could improve the compressive strength up to 10%, but a 0.30% CF dosage could adversely affect the compressive strength significantly, which was due to the air entrainment [68]. Likewise, the 0.15% CF dosage could improve the flexural strength by about 10%, but the 0.30% CF dosage could only improve the flexural strength marginally (6%).

#### 3.4.4. Microstructure

**Figure 10:**
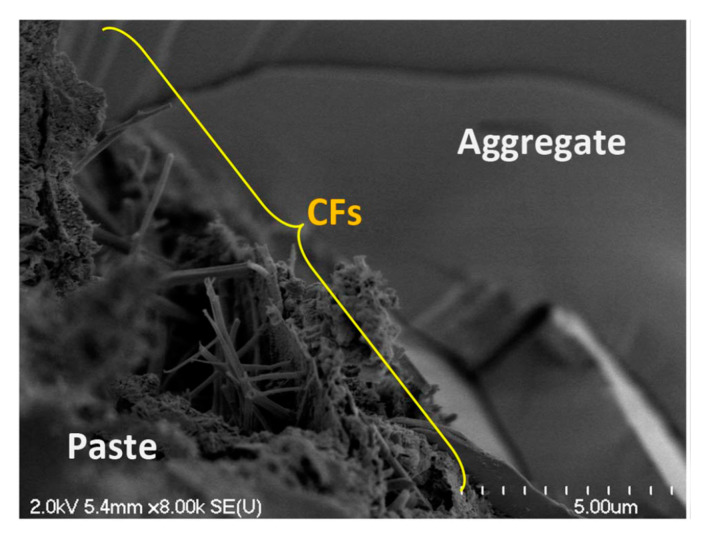
SEM image of the interfacial transition zone (ITZ) in self-consolidating concrete (SCC) with 0.15% CFs. Reproduced from [21], with permission from American Society of Civil Engineers, 2018.

The microstructure analysis showed that for cement pastes, CFs could adhere to the pastes well [21]. For self-consolidating concrete (SCC), CFs could reinforce the interfacial transition zone (ITZ) between cement paste and aggregates, as shown in Figure 10 [21]. The micromechanical properties (indentation modulus, indentation hardness, and contact creep modulus) of the C-S-H gel matrix were improved by CFs, which was attributed to a combination of a higher degree of hydration at a given age and the reinforcing effect caused by CFs [26].

#### 3.4.5. Rheology

The addition of CFs at dosages of 0.1%, 0.15%, and 0.2% by weight of binder could increase the yield stress of paste mixture significantly, which was due to the hydrophilic nature of CFs, leading to water absorption, and also due to the high aspect ratio and flexibility of CFs, leading to the formation of filament networks [21]. The feasibility of using CFs as a viscosity-modifying agent (VMA) in self-consolidating concrete (SCC) was explored, and it was found that CFs could improve the mixture stability by increasing the yield stress and the plastic viscosity of SCC, indicating a significant viscosity-modifying effect [11]. In addition, the mixture with CFs exhibited a shear-thinning effect, which allowed it to have a high apparent viscosity at a low shear rate that is necessary for stability and have a low apparent viscosity at a high shear rate that is necessary for improving pumpability.

#### 3.4.6. Shrinkage and Durability

The effect of CFs on the shrinkage and durability of cementitious materials cannot be found in the existing research. Future studies are needed on these topics.

### 3.5. Challenges of Applying Nanocelluloses in Cementitious Materials

Although nanocellulose (in different forms) can improve the fresh properties, mechanical properties, volume stability, and durability, the effectiveness depends on material processing and dispersion. One should notice that the biggest challenge related to the application of nanocellulose in cementitious materials is the agglomeration issue. Typically, the nanocellulose can be dispersed using the surfactant agent (e.g., water reducing agent (WRA)) and the sonication method. However, it was reported that the WRA was not effective as a surfactant in dispersing CNCs, because the flexural strength of samples with WRA did not show greater improvement compared to those without WRA [28]. For the sonication method, it was reported that the size of CNC agglomerates could be decreased from 14.7 to 2.23 μm if the sonication time was increased from 0 to 10 min, with a low-frequency ultrasound of 20 kHz and an amplitude of 60% [69]. Even a short 1 min sonication was very effective in dispersing large aggregates, leading to considerably smaller particles with a median size of 4.3 μm. Improving the dispersion of nanocellulose is advantageous to the performance of cementitious materials. Cao et al. suggested that the flexural strength of cement pastes with sonicated CNCs was improved compared to cement pastes with unsonicated CNCs [28]. To successfully implement the sonication procedure for dispersing nanocellulose in cementitious materials, some critical points are highlighted as follows.

**Figure 11:**
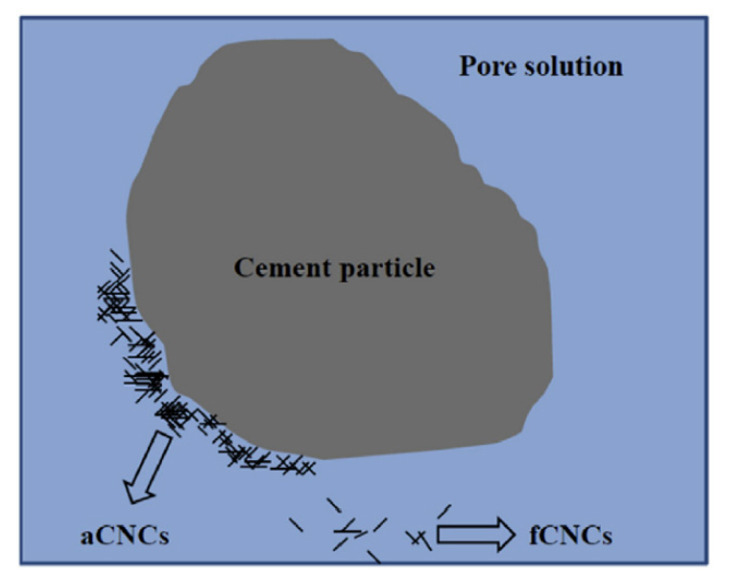
Schematic illustration of two different types of CNCs in cement paste. Reproduced from [58], with permission from Elsevier, 2016.

The zeta-potential test indicated that CNCs or CNFs tended to adhere to cement particles rather than to agglomerate themselves [14,50]. Therefore, when CNCs or CNFs are added to cementitious materials, many of them can be absorbed onto cement particles. Cao et al. categorized CNCs into two types, aCNCs (absorbed on the surface of cement particles) and fCNCs (free in water), as shown in Figure 11 [58]. It was reported that more than 90% CNCs exist in the form of aCNCs, and even though sonication can disperse CNC agglomerates, it cannot help aCNCs convert into fCNCs. Therefore, the sonication mainly contributes to disperse the agglomerates of nanocellulose.

Normally, when the nanocellulose concentration is below a critical concentration, the nanocellulose is mostly in the form of free particles; beyond the critical concentration, the nanocellulose can easily form aggregates or network structures. A commonly used way of dispersing nanocellulose is mixing nanocellulose with water, and the sonication is applied to the nanocellulose solution to reduce agglomerates. Then the sonicated nanocellulose solution is added to the cementitious materials. However, it should be noted that even if the nanocellulose is dispersed well in water by sonication, it may not necessarily be dispersed well in cementitious materials. In the cementitious materials, ionic species such as K^+^, Na^+^, Ca^2+^, OH^−^, and SO^4−^ exist in the pore solution and are likely to change the surface charges on nanocellulose to make nanocellulose adhere to each other and form more and larger agglomerates. Cao et al. found that compared to CNCs in water, the critical concentration was reduced from 1.35 to 0.18 vol% when the CNCs were dispersed in a simulated cement pore solution [28]. Therefore, it can be found that even if nanocellulose in water is sonicated well, with little agglomerates, more agglomerates may form in cementitious materials.

Some caution should be taken while sonicating nanocellulose because the sonication amplitude and duration can significantly affect the properties of nanocellulose. It was reported that the increase of sonication amplitude or time could decrease the length, width, and aspect ratio of CNCs and even destroy the crystalline structure of CNCs [69,70]. Therefore, a proper sonication amplitude and time (sonication energy) is very important for dispersing nanocellulose. Normally, a higher sonication energy per unit mass of nanocellulose is expected to result in better dispersion of nanocellulose. However, even if the sonication energy per unit mass of nanocellulose remains constant, the size and size distribution of nanocellulose can vary with the concentration of nanocellulose suspension [71]. Therefore, in addition to a proper sonication energy, a proper concentration of CNC suspension is also important for better dispersion.

## 4. Conclusions

Four types of nanocellulose (CNC, CNF, BC, and CF) have been used in cementitious materials by some researchers, and their effects on physical and fresh properties, hydration, mechanical properties, microstructure, rheology, shrinkage, and durability of cementitious materials are reviewed. Based on the existing research, some important conclusions can be drawn.

For cementitious materials with CNCs, it was found that CNCs can increase density and reduce water absorption of cementitious materials; CNCs can delay the initiation of cement hydration but increase the degree of hydration at a given age; a modest dosage of CNCs can improve the mechanical properties, whereas a high dosage of CNCs can result in a negative effect; CNCs can reduce the porosity and refine the pore structure; the drying shrinkage can be reduced; when the CNC dosage is below a specific threshold, CNCs can reduce the yield stress, and beyond this threshold, CNCs can increase the yield stress; the carbonation depth and chloride ion penetration can be reduced, whereas there is some controversy about the freezing-thawing resistance.

For cementitious materials with CNFs, it was found that CNFs can reduce the flowability, which can be used as viscosity-modifying agents, and also increase density, which can help reduce water absorption but increase thermal conductivity and thermal expansion; CNFs can delay the initiation of cement hydration but increase the degree of hydration at a given age; a modest addition of CNFs can increase the mechanical properties and yet too many CNFs can lead to an opposite result; CNFs can refine the pore size, but there is no consistent conclusion in terms of the porosity; the free shrinkage and autogenous shrinkage can be reduced by using CNFs; the yield stress and the plastic viscosity can be improved; CNFs can reduce sulfate and chloride ion penetration, and increase freezing-thawing resistance.

For cementitious materials with BCs, it was found that BCs can reduce water absorption, improve mechanical properties, and serve as nano-bridges to arrest nano-cracks. The effect of BCs on the hydration, shrinkage, rheology, and durability of cementitious materials cannot be found in the existing studies.

For cementitious materials with CFs, it was found that CFs can reduce the flowability and increase air content; the degree of hydration at a given age can be increased; the mechanical properties can be improved; CFs can reinforce the interfacial transition zone (ITZ) between cement paste and aggregate; CFs can also increase the yield stress and serve as viscosity-modifying agents. The effect of CFs on the shrinkage and durability of cementitious materials cannot be found in the existing studies.

Generally, it can be concluded that the nanocellulose type, nanocellulose dosage, nanocellulose dispersion, and the matrix type can affect the properties of cementitious materials significantly. Therefore, while using nanocellulose in cementitious materials, all these related factors should be examined in detail.

The biggest challenge of applying nanocellulose in cementitious materials is the dispersion issue. Sonication is the most commonly used method for dispersing nanocellulose, of which three critical points need to be considered for its successful implementation. The first is that the sonication can disperse nanocellulose but can hardly convert the nanocellulose absorbed on cement particles into free nanocellulose. The second is that even if nanocellulose in water can be dispersed well by sonication, more agglomerates may still form in cementitious materials. The third is that a proper sonication energy and nanocellulose suspension concentration need to be established.

In addition, it can be seen that compared to other nanomaterials, the studies on the cementitious materials with nanocellulose are still very limited. On the one hand, there are still many topics (not limited to the topics covered in this review) that need to be explored in future studies; on the other hand, there are some controversies in some specific topics from the existing studies, and more studies are needed to provide strong supports for drawing any conclusions.

## Figures and Tables

**Table 1:** Mechanical properties of cementitious materials with CNCs.

Cementitious Materials	CNC Source	CNC Dosage	Mechanical Properties	Ref.
Type I Portland cement mortar	Manufactured from α-cellulose	0.2, 0.4, 0.6, and 0.8% by volume of cement	Increase compressive strength and flexural strength, with 0.4% dosage exhibiting the best	[15]
Concrete made with Portland cement CEM I 32.5R	Manufactured from carrot cellulose	0.5 and 1% by weight of cement	Increase flexural strength and compressive strength, with 0.1% dosage exhibiting better	[27]
Type I Portland cement paste	NA	0.2 and 1% CNC by volume of cement paste	Increase compressive strength; 0.2% dosage increase the peak load during three-point bending test, whereas 1% decrease the peak load	[51]
Fiber-reinforced ordinary Portland cement paste	“C” company in Canada	0.4, 0.8, and 1.2% by volume of cement	Increase compressive strength and flexural strength, with 0.8% as the optimal dosage	[13]
Fiber-reinforced high-toughness Ordinary Portland cement composites (ECC)	Manufactured by a chemical process from wood-based cellulose	0.4, 0.8, and 1.2% by volume of cement	Increase compressive strength and flexural strength, with 0.8% as the optimal dosage	[53]
High early strength cement (Votoram), by Brazilian Standards NBR 16697	CelluForce company	0.1, 0.2, 0.5, and 1% by volume of cement	0.1–0.5% dosage can increase the dynamic modulus of elasticity at an early age (up to 14 days) but cannot have a significant influence at 28 days; 1% dosage can hardly result in any improvement at all ages from 3 days to 28days	[49]
Oil well cement paste	Canada’s 2nd largest manufacturing plant in Edmonton	Approximately 0.5% by weight of cement	Increase compressive strength, tensile strength, storage modulus, and loss modulus but reduce damping factor	[56]
Calcium aluminate cement (CAC) paste and ordinary Portland cement (OPC) paste	Acid hydrolysis on crystalline microcellulose (Sigma-Aldrich)	0.1, 0.2, 0.4, and 0.8% by weight of cement	In CAC paste, 0.1% and 0.2% dosages increase the modulus of rupture and modulus of elasticity, but 0.4% and 0.8% dosages result in the opposite effect; in OPC paste, the improvement is not observed.	[54]
Type V Portland cement paste	USDA Forest Service-Forest Products Laboratory, Madison, WI	0.04, 0.1, 0.2, 0.5, 1.0, and 1.5% by volume of cement	At 3 days, the flexural strength increases with increasing dosage, while for older ages, it reaches a peak at 0.2% dosage and then decreases	[50]

**Table 2:** Mechanical properties of cementitious materials with CNFs.

Cementitious Materials	CNF Source	CNF Dosage	Mechanical Properties	Ref.
Concrete made with Portland cement CEM I 32.5R	Manufactured from apple cellulose	0.5 and 0.1% by weight of cement	Increase flexural strength and compressive strength, with 0.1% dosage exhibiting better strength	[27]
Calcium aluminate cement (CAC) paste and ordinary Portland cement (OPC) paste	A high-intensity mechanical refining process on sisal pulp	0.1, 0.2, 0.4, and 0.8% by weight of cement	In CAC paste, 0.1% dosage increases the modulus of rupture and modulus of elasticity, but 0.2–0.8% dosage results in the opposite effect; in OPC paste, the improvement is not observed	[54]
Type I/II Portland cement paste	University of Maine Process Development	0.015, 0.030, 0.060, 0.090, and 0.150% by weight of cement	All dosages can improve compressive strength and especially flexural strength	[14]
Concrete and paste made with Type I/II Portland cement	University of Maine Process Development	0.05, 0.1, 0.2, 0.5, 1.0, 1.5, and 3.0% by volume of cement for paste; 0.1, 0.2, and 0.3% by volume of cement for concrete	For paste (w/c = 0.35), 0.05–0.2% dosage can increase compressive strength, and if w/c > 0.35, no clear improvement is observed; 0.5–3.0% dosage decreases compressive strength. For concrete, all dosages reduce compressive strength	[19]
Type GU Portland cement mortar	Sourced commercially and manufactured at the University of Alberta	0.1, 0.2, 0.3, 0.4, and 0.5% of dry CNF by volume of mortar	0.1–0.5% decreases compressive strength to a modest extent; no significant difference is observed between the CNF variants	[66]
CEM I 32.5 N Portland cement paste	Manufactured from commercial bleached eucalyptus pulp (Eucalyptus globulus)	0.01, 0.05, 0.1, 0.2, 0.3, and 0.5% by weight of cement	0.01–0.3% dosage enhances compressive strength; 0.5% dosage reduces compressive strength dramatically below that of plain paste	[61]
Ordinary Portland cement (PII 42.5 R) paste	Isolated from wood	0.05, 0.1, 0.15, 0.25, and 0.4% by weight of cement	3-day compressive and flexural strengths are not affected obviously; 28-day strengths are improved significantly by 0.05–0.15% dosage but are reduced if the dosage is beyond 0.15%	[60]
Type H oil well cement paste	Acid hydrolysis on W-50 grade bleached wood pulp	0.04, 0.12, 0.20, and 0.28% by weight of cement	0.04% dosage increases flexural strength significantly; beyond this dosage, the flexural strength is reduced	[63]
Type H oil well cement paste	Acid hydrolysis on W-50 grade bleached wood pulp	0.04% by weight of cement	No increase in compressive strength is observed; flexural strength is improved significantly	[64]

**Table 3:** Mechanical properties of cementitious materials with BCs.

Cementitious Materials	BC Source	BC Dosage	Mechanical Properties	Ref.
Ordinary Portland cement (GEM I-32.5) mortar	Made using *Gluconacetobacter xylinus*	0.1, 0.3, and 0.5% by weight of cement	Increase flexural strength and compressive strength while the optimum dosage varies with BC form	[20]
Type II Portland cement mortar	Made using *Gluconacetobacter xylinus*	NA	Increase modulus of rupture, modulus of elasticity, and fracture toughness	[45]

**Table 4:** Mechanical properties of cementitious materials with cellulose filaments (CFs).

Cementitious Materials	CF Source	CF Dosage	Mechanical Properties	Ref.
Paste and self-consolidating concrete (SCC) made with Type general use (GU) cement	Provided by Kruger Biomaterials Inc.	0.1, 0.15, and 0.2% by weight of binder (fly ash+cement)	For paste, all dosages reduce compressive strength but increase flexural strength; for concrete, all dosages increase both flexural and compressive strengths	[21]
Type general use (GU) cement paste	Provided by Kruger Biomaterials Inc.	0.05, 0.10, 0.20, and 0.30% by weight of cement	Improve compressive and flexural strengths, elastic modulus, and toughness	[26]
Strain-hardening cementitious composites made with Type high-sulfate-resistance (HS) cement	Provided by Kruger Biomaterials Inc.	0.03, 0.05, and 0.1% by weight of cement	Improve ultimate strain capacity (in uniaxial tension) and deflection capacity (in flexure), resulting in better ductility	[12]
Ultra-high performance concrete made with Type high-sulfate-resistance (HS) cement	Provided by Kruger Biomaterials Inc.	0.15 and 0.3% by weight of cement	0.15% dosage increases compressive and flexural strengths; 0.3% dosage results in an opposite result	[68]
